# Next Generation Sequencing As an Aid to Diagnosis and Treatment of an Unusual Pediatric Brain Cancer

**DOI:** 10.3390/jpm4030402

**Published:** 2014-07-15

**Authors:** John Glod, Mihae Song, Archana Sharma, Rachana Tyagi, Roy H. Rhodes, David J. Weissmann, Sudipta Roychowdhury, Atif Khan, Michael P. Kane, Kim Hirshfield, Shridar Ganesan, Robert S. DiPaola, Lorna Rodriguez-Rodriguez

**Affiliations:** 1 Rutgers Cancer Institute of New Jersey, 195 Little Albany Street, New Brunswick, NJ 08903, USA; E-Mails: john.glod@nih.gov (J.G.); sharmaar@cinj.rutgers.edu (A.S.); khanat@cinj.rutgers.edu (A.K.); kanemp@cinj.rutgers.edu (M.P.K.); hirshfie@cinj.rutgers.edu (K.H.); ganesash@cinj.rutgers.edu (S.G.); dipaolrs@cinj.rutgers.edu (R.S.D.); 2 Rutgers Robert Wood Johnson Medical School, 125 Paterson Street, New Brunswick, NJ 08901, USA; E-Mails: songm2@rwjms.rutgers.edu (M.S.); tyagira@rwjms.rutgers.edu (R.T.); 3 Rutgers Robert Wood Johnson Medical School, 1 Robert Wood Johnson Place-MEB 212, New Brunswick, NJ 08903, USA; E-Mails: rhodesrh@rwjms.rutgers.edu (R.H.R.); weissmdj@rwjms.rutgers.edu (D.J.W.); 4 University Radiology Group, 579-A Cranbury Road, East Brunswick, NJ 08816, USA; E-Mail: sr10@comcast.net

**Keywords:** glioblastoma, pediatric glioma, metastatic glioma, gene mutation, next generation sequencing, BRAF V600E, vemurafenib, CKDN2A

## Abstract

Classification of pediatric brain tumors with unusual histologic and clinical features may be a diagnostic challenge to the pathologist. We present a case of a 12-year-old girl with a primary intracranial tumor. The tumor classification was not certain initially, and the site of origin and clinical behavior were unusual. Genomic characterization of the tumor using a Clinical Laboratory Improvement Amendment (CLIA)-certified next-generation sequencing assay assisted in the diagnosis and translated into patient benefit, albeit transient. Our case argues that next generation sequencing may play a role in the pathological classification of pediatric brain cancers and guiding targeted therapy, supporting additional studies of genetically targeted therapeutics.

## 1. Introduction

Brain tumors are the most common solid malignancy of childhood [1,2]. Surgical resection, radiation therapy and chemotherapy are critical parts of the initial treatment strategy for high-grade, pediatric brain tumors [3,4]. However, there are limited effective strategies for recurrent/resistance disease. As our knowledge of the molecular drivers of these resistant tumors expands, genetic aberrations that can be therapeutically targeted may be identified [3,5,6].

Systemic metastasis of primary central nervous system tumors is an uncommon event [7,8,9]. We report a young patient with a primary intracranial tumor with extracranial metastatic spread to the temporalis muscle adjacent to the operative site, cervical lymph nodes and axial bone and bone marrow. The tumor classification was not certain initially, and the site of origin and clinical behavior were unusual. Molecular characterization of the tumor demonstrated a well-described BRAF V600E point mutation and loss of *CDKN2A*/*B*. Both mutations have been reported in high-grade pediatric gliomas [6,10,11]. This case illustrates the utility of molecular analysis in both the diagnosis and identification of potential treatment options in pediatric cancers.

## 2. Case Presentation

**Figure 1:**
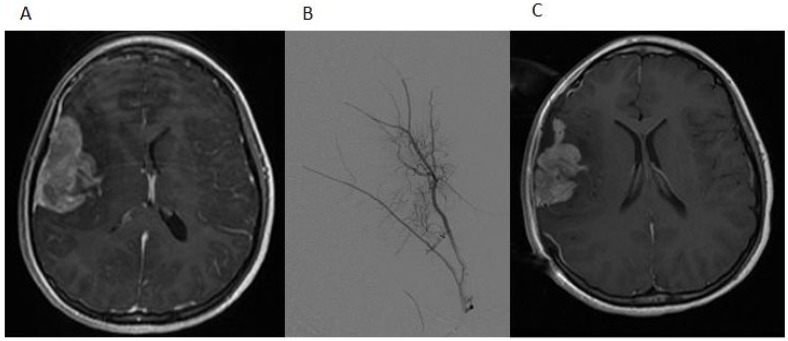
(**A**) The post-contrast T1-weighted MRI image shows a predominantly heterogeneously enhancing mass extending into the right Sylvian fissure with probable invasion of the adjacent insular cortex; (**B**) a select image from the tumor embolization demonstrates a dramatically hypervascular tumor supplied by branches of the right middle meningeal artery that was successfully embolized with polyvinyl alcohol particles; (**C**) the postoperative T1-weighted MRI image with contrast demonstrates partial resection of the tumor.

A 12-year-old girl presented with a history of frontal headaches for several weeks after suffering minor head trauma. The initial CT scan without contrast showed no evidence of intracranial bleeding. Because of persistent headaches, however, as well as nausea and vomiting, she underwent another CT scan less than a month later that showed a 6.2 × 3.5 cm right fronto-temporal mass with associated edema and a midline shift. Magnetic resonance imaging (MRI) of the brain with and without intravenous gadolinium was then performed and demonstrated a 5.5 × 2.2 × 5.1 cm, apparently extra-axial mass, largely within the right Sylvian fissure (Figure 1A). The interface of the brain was lobulated and irregular, raising the possibility of the invasion of the brain and subarachnoid space, but no definite intraparenchymal component was recognized. There was vasogenic edema in the subcortical white matter and a 5-mm midline shift towards the left side. Based on imaging, it was felt that the tumor was likely to be a hemangiopericytoma. Physical examination was notable for an intact neurological examination.

**Figure 2:**
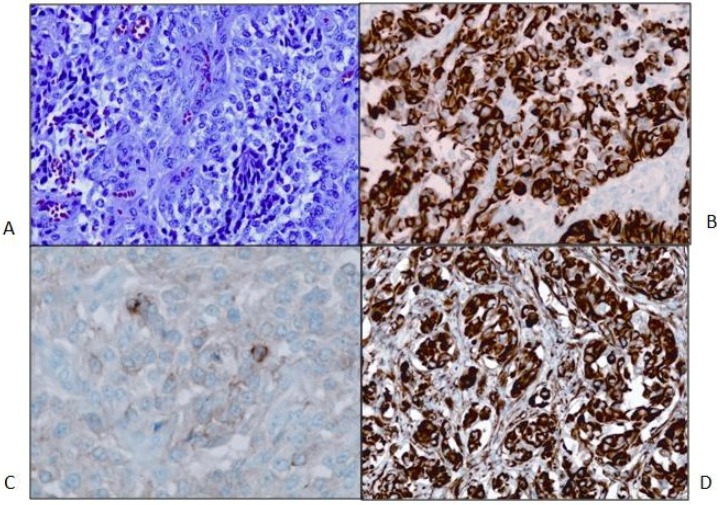
(**A**) Primary tumor. H&E stain. 200× magnification. The neoplasm is relatively cellular and formed of crowded polygonal cells with variable amounts of pale eosinophilic or clear cytoplasm. Nuclei are round to oval and often irregular, and they are vesicular with occasional small nucleoli. Binucleated tumor cells are present. Mitotic figures are rare. A few small tumor cells have slightly expanded cytoplasm with fibrous inclusions (nonspecific rhabdoid cells). Thin fibrovascular septa, mostly with capillary-size vessels, are present, forming vague small lobules in many areas; (**B**) Primary tumor. GFAP stain; 200× magnification; (**C**) Primary tumor. Synaptophysin stain; 400× magnification; (**D**) Primary tumor. Nestin stain of tumor; 200× magnification.

The patient underwent a right-sided craniotomy after a successful partial embolization of the tumor the day prior (Figure 1B). A significant portion of the mass was visualized, deep to middle cerebral artery branches shown by intraoperative ultrasound to be coursing through the mass and providing blood supply to the temporal and frontal lobes. The attempted dissection of the tumor off the pial surface also led to devascularization of the underlying brain as the tumor had parasitized the pial vasculature. Therefore, further dissection following superficial excisional biopsy of the mass was not performed for safety reasons. A dural graft was placed. Because of significant brain swelling, the bone flap was not immediately replaced. The temporalis muscle was closed in apposition to the dural graft. Postoperative MR imaging showed that the resection of the anterior and superior aspects of the mass had been accomplished, although the majority of the mass remained (Figure 1C). Microscopic examination revealed a cellular neoplasm with clear or eosinophilic cytoplasm in sheets, small nests and pseudopapillary formations associated with small foci of necrosis (Figure 2A). There were rare mitotic figures. Thin connective tissue bands with blood vessels containing endothelial hyperplasia were frequent. Immunostaining revealed vimentin in all of the tumor cells and scattered tumor cells positive for bcl-2, p53, desmin and factor XIIIa. CD99 and CD34 were negative. The mitotic labeling index using Ki-67 staining was approximately 10%. The initial pathologic diagnosis was hemangiopericytoma.

**Figure 3:**
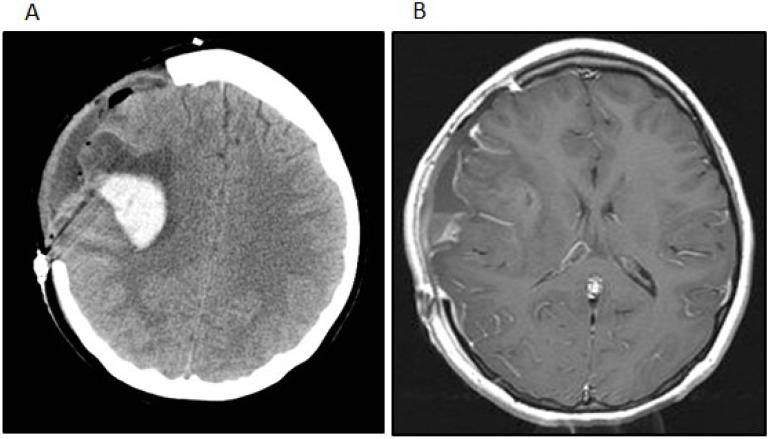
(**A**) The patient developed a delayed intratumoral hemorrhage requiring hemicraniectomy and evacuation of the hemorrhage and tumor. The noncontrast head CT shows the acute hemorrhage within the right Sylvian fissure extending into the right frontal lobe; (**B**) The post-contrast t1-weighted image after the second surgery shows subtotal removal of the neoplasm and evacuation of the intratumoral hemorrhage.

Postoperatively, the patient remained neurologically intact. One month after her initial surgery, however, the patient developed a rapidly worsening mental status that progressed to coma. Imaging studies showed a hemorrhage within and an interval growth of the tumor (Figure 3A). The patient underwent an emergent craniotomy with the evacuation of the hemorrhage and near total resection of the residual tumor (Figure 3B). Although the patient experienced a complete recovery from a cognitive standpoint, she did have a severe left-sided hemiparesis. Two months after the emergent surgery, she underwent another craniotomy for the resection of the recurrent tumor and replacement of her bone flap in preparation for radiation therapy. At that time the tumor was noted to be densely adherent to the overlying dura and temporalis muscle, from which all gross tumor was removed. The resected tumor revealed a few minute areas of infiltration into superficial cortex with no bulk tumor identified within the brain. However, areas of dural infiltration and some infiltration of the temporalis muscle were identified.

Second opinions on the pathologic diagnosis with additional immunohistochemical staining revealed that all of the tumor cells were positive for glial fibrillary acidic protein (GFAP). A diagnosis of high-grade glioma was made. Findings of astrocytic tumor cells, vascular endothelial hyperplasia and focal necrosis, along with accumulating imaging and intraoperative information, led to the conclusion that the tumor was a glioblastoma multiforme (GBM) that might have initially expanded in the subarachnoid space rather than subcortically. Further studies included immunostaining for nestin with all tumor cells being positive and for synaptophysin showing focally positive tumor cells from all three resections with slightly increased staining in the second tumor sample and larger foci of stained cells in the last resection (Figure 2B,D). E-cadherin was negative in the initial tumor biopsy, while there was focal membrane staining in the tumor from the third craniotomy. The patient was treated with external beam radiation therapy 60 Gy with concomitant temozolomide at a dose of 75 mg/m^2^/day during radiation. Over the course of radiation therapy, she remained clinically stable.

**Figure 4:**
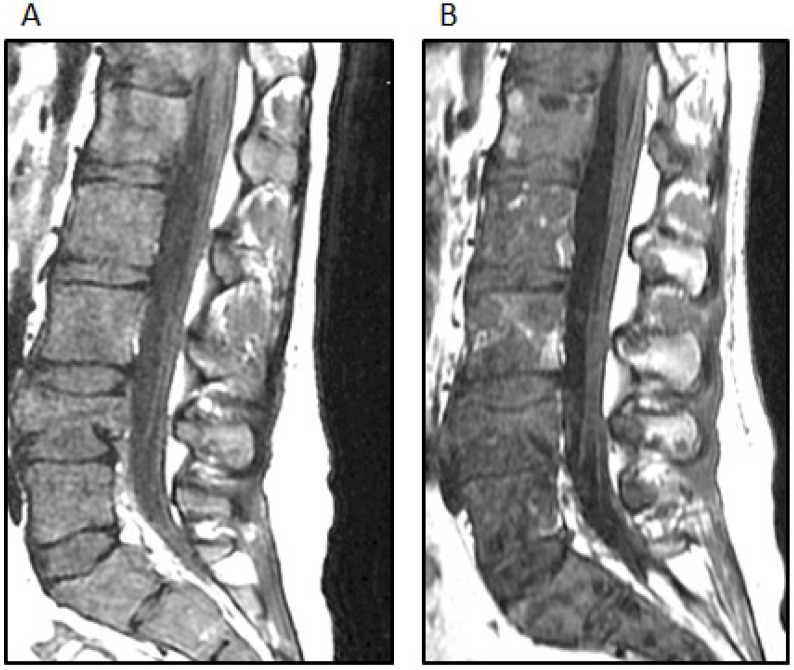
(**A**) The sagittal STIR (short TI inversion recovery) MRI image demonstrates extensive new osseous metastases with multiple pathologic compression fractures of the lumbar spine; (**B**) The sagittal T-weighted MRI image demonstrates interval mild improvement in bone marrow signal abnormality after treatment; the fatty replacement of tumor is shown by white arrows.

**Figure 5:**
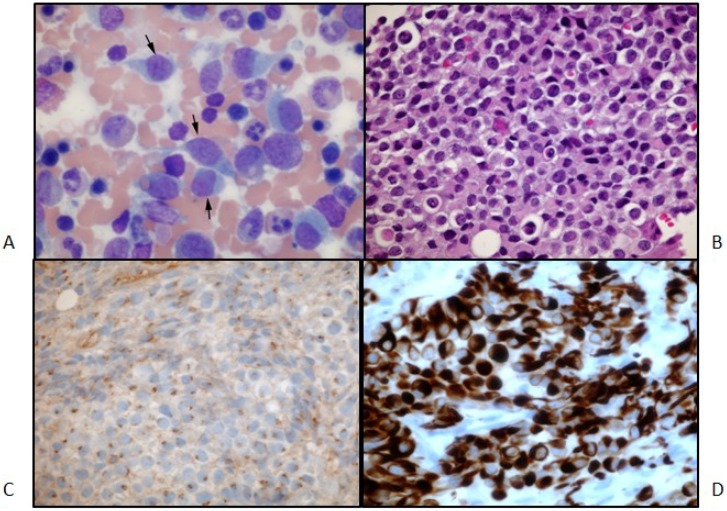
(**A**) Bone metastases aspirate smear. Wright-Giemsa stain; 600× magnification. The arrows point to some of the malignant cells in this field; (**B**) Bone metastases, high power: bone marrow biopsy. Hematoxylin and eosin stain; 400× magnification. A sheet of malignant cells at high magnification; (**C**) Bone metastases, synaptophysin stain: bone marrow biopsy. Immunohistochemical stain for synaptophysin; 400× magnification. A stain for synaptophysin shows peri-nuclear dot-positivity in the malignant cells; (**D**) Bone metastases, GFAP stain: bone marrow biopsy. Immunohistochemical stain for GFAP; 400× magnification. A stain for GFAP marks the cytoplasm of the malignant cells.

Two weeks after the completion of radiation therapy, the patient developed painless bilateral cervical adenopathy with several firm, immobile, right posterior cervical lymph nodes approximately 2 cm in the greatest diameter. One week later, the patient developed midline back pain primarily in the lumbar area and also pancytopenia. An MRI demonstrated the bilateral cervical adenopathy, as well as lytic vertebral lesions of the cervical, thoracic and lumbar spine with multifocal bone marrow involvement (Figure 4A). A mass deep to the right sternocleidomastoid muscle was excised. Microscopically, there was an infiltrated lymph node along one edge, and the mass consisted of sheets of poorly-differentiated metastatic tumor cells positive for GFAP and rarely for synaptophysin. A right iliac crest bone marrow biopsy was performed, and it showed 60% infiltration with GFAP-positive and nestin-positive tumor cells, while synaptophysin was focally positive (Figure 5).

Since the tumor had progressed with extracranial metastases on temozolomide, the patient was started on treatment with vincristine, dactinomycin and cyclophosphamide, as well as external radiation therapy to the lower spine and hip. Because of the unusual course of the disease, a metastatic tumor of the neck was tested for molecular alterations, using a CLIA certified targeted resequencing panel of “actionable” genes using hybrid-capture and next-generation sequencing approaches [12,13]. Briefly, formalin-fixed, paraffin embedded tumor specimens were sent to have targeted sequencing performed on 236 genes and 47 introns of 19 genes that are involved in fusions. The alterations found in this tumor were the BRAFV600E mutation, CDKN2A loss and PTPRD S1845fs*2. Allele frequencies for BRAF and PTPRD were 64% for BRAF, with a read depth of 2,838; 70% for PRPRD at a read depth of 1,504. The tumor purity was estimated at 60% computationally.

This pattern of mutations has been reported in high-grade pediatric gliomas and supported this diagnosis. The presence of a BRAF V600E mutation suggested additional therapeutic options. Following the discussion of this case at the Rutgers Cancer Institute of New Jersey Molecular Tumor Board, vemurafenib (960 mg BID), an oral BRAF inhibitor, was added to her treatment regimen. She developed a macular rash on the extremities five days after starting vemurafenib, while both back pain and pancytopenia improved and the cervical adenopathy regressed. An MRI of the spine also showed partial regression of the bone marrow disease (Figure 4B). After four weeks of this treatment, she developed worsening back and hip pain, and the size of the cervical adenopathy increased. Her chemotherapy regimen was changed to carboplatin, which did not result in a sustained response. Comfort care was pursued, and the patient subsequently died of her disease, 11 months after symptoms began and 10 months after the initial craniotomy. No autopsy was performed.

## 3. Discussion

The tumor unexpectedly contained a BRAF V600E mutation, CDKN2A loss and PTPRD S1845fs*2 mutation. This case illustrates that in the treatment of pediatric oncology patients with an atypical clinical course, molecular data may play a role both in tumor classification and in the identification of therapeutic options. While overall gene expression is qualitatively comparable in congenital, pediatric and primary adult GBM [14], the frequency of common genetic alterations differ. In pediatric diffuse astrocytomas (WHO Grades 2 to 4, including GBM), there is a lower frequency of the genetic alterations most commonly observed in adult cases [15]. An exception exists for a specific point mutation of the *v-raf* murine sarcoma viral oncogene homolog B1 (*BRAF*). The *BRAF* gene is the second most common gene mutated in human cancers [16]. The BRAF V600E point mutation has been identified in 6% of pediatric GBM, while it has not been identified in adult GBM [17]. Conversely, homozygous deletion in *CDKN2A* occurs at a lower rate in pediatric GBM than in adult cases [15]. The *CDKN2A* tumor suppressor genes appear to contribute to glioma predisposition, both from common single nucleotide polymorphisms and from rare mutations. Their loss carries a poor prognosis [18]. A subset of high-grade pediatric astrocytomas into which our patient fits has a BRAF mutation and *CDKN2A* inactivation [12,15]. The combination of BRAF V600E mutation and *CDKN2A* deletion leads to reduced differentiation in murine neural progenitor cells [12].

Some pediatric astrocytomas lacking the BRAF V600E mutation contain *KRAS* or other mutations in pathways that increase BRAF activity [17]. BRAF V600E mutations are found in small subsets of some low-grade gliomas, including cerebellar and non-cerebellar pilocytic astrocytomas, pleomorphic xanthoastrocytomas and gangliogliomas [12,17]. Patients with low-grade gliomas and the BRAF V600E mutation have poorer outcomes and a decreased progression-free survival [12].

The prevalence of the BRAF V600E mutation in several solid tumors led to the development of its specific inhibitor, vemurafenib. Although there is very little experience using vemurafenib in children, it has shown significant activity against BRAF V600E mutation-positive metastatic melanoma in adults. Eighty percent of patients with metastatic melanoma had either partial or complete remission, and 74% had a reduction in disease progression or mortality [19]. Vemurafenib is well tolerated, with major side effects including fatigue and photosensitivity. Unfortunately, the response to vemurafenib in melanoma is often transient. The combination of BRAF inhibitor and MEK inhibitors may be more effective than BRAF inhibitors alone.

Murine models of BRAF V600E mutant gliomas suggest that BRAF inhibitors may be active in this setting [20,21]. A recent study using a glioma model in which there is concomitant BRAF V600E mutation and CDKN2A loss, as seen in our patient, demonstrated that these tumors respond to asingle-agent BRAF inhibitor [13]. However, much better responses were seen with the combination of BRAF inhibitor and CDK 4/6 inhibitor. These data suggest that combination therapy targeting both BRAF and CDK 4/6 may be useful in high-grade gliomas harboring BRAF V600E and CDKN2A loss [13]. Unfortunately no clinical trials, open to pediatric patients, testing BRAF inhibitors, MEK inhibitors or CDK4/6 inhibitors were available for our patient. She was treated with vemurafenib, as it was the only appropriate agent targeting BRAF V600E that was FDA approved at the time, and she had a transient response. Use of BRAF inhibitors in pediatric brain cancers has been reported in a case report of pediatric brain stem ganglioglioma with a BRAF V600E mutation, where the patient was successfully treated with vemurafenib and vinblastine [22].

## 4. Conclusions

The current case demonstrates the importance of the use of clinical genomic sequencing to identify molecular alterations that drive tumor development, especially in rare, pathologically ambiguous cancers. This case also emphasizes that genetic alterations should be determined early rather than late in the diagnostic process to aid with both diagnosis and treatment decisions. If genomic sequencing information had been present earlier in the course of this patient, there might have been more time to develop more sophisticated targeted treatment options. In this era of personalized medicine, molecularly targeted agents developed to inhibit specific oncogenic pathways offer more treatment options that may decrease recurrence and prolong survival. Clinical trials are needed to investigate optimal targeted therapy for BRAF V600E mutant pediatric brain cancers.
